# Pathways and Barriers to Careers in Academic Clinical Cancer Prevention: a Qualitative Study

**DOI:** 10.1007/s13187-020-01921-z

**Published:** 2020-11-12

**Authors:** Melissa Y. Kok, Janelle C. Chavez, Pompeyo R. Quesada, Oluwapelumi T. Adegoke, Shine Chang

**Affiliations:** 1 Baylor College of Medicine, Houston, TX USA; 2 Stanford University School of Medicine, Stanford, CA USA; 3 Otolaryngology-Head and Neck Surgery, The University of Kansas Medical Center, Kansas City, KS USA; 4 Department of Psychiatry and Behavioral Health, Virginia Tech Carilion School of Medicine, Roanoke, VA USA; 5 Department of Epidemiology, Division of Cancer Prevention and Population Sciences, The University of Texas MD Anderson Cancer Center, Houston, TX USA

**Keywords:** Occupational choices, Training, Professional development, Medicine

## Abstract

National surveys document steady declines over time in interest in academic medicine and cancer prevention careers (Am J Prev Med 54(3):444–8, 2018). Through interviews with 16 academic cancer prevention physicians at one comprehensive cancer center, this study identifies motivations and barriers to physician careers in academic cancer prevention and proposes recommendations to increase recruitment. Participants reported that cancer prevention was vague to them early in training, impairing career exploration. Further, without role models and opportunities to learn about cancer prevention, many were ignorant of career options. Many had incorrect views about cancer prevention practice being mainly within the scope of primary care physicians, and some reported colleagues viewing the rigor of cancer prevention skeptically. However, all described notable experiences—in classes, with mentors, on research projects, or from encounters with patients, motivating them to pursue academic clinical cancer prevention regardless of challenges. Clearly, a lack of both information and guidance towards careers in clinical cancer prevention has been critical barriers to robust recruitment of physicians to the field and must be addressed urgently. Helping physicians earlier during training to both understand the value of prevention and cultivate their interests in it, particularly for clinical cancer prevention, would have widespread benefits.

## Introduction

Advances against cancer, particularly in preventing cancers, can only result from translating research discoveries into practice in the clinic and community, often led by physicians. Despite the critical role that physicians play in furthering cancer prevention, interest in this field has declined [1]. A national survey in 2014 found that < 10% of oncology fellows intended to focus on cancer prevention [2], suggesting that among physicians choosing careers in cancer, the number who will feature cancer prevention in their careers will be small. Further, current recruitment rates to oncology are lower than retirement rates among cancer prevention physicians in the USA, indicating that the number of physicians in cancer prevention will continue to shrink unless deliberate efforts are made [1].

Studies reporting low recruitment rates generally cite as contributing factors, a lack of clarity about pathways into careers in clinical cancer prevention [2]. Lack of clarity could relate to the field of cancer prevention being broad, encompassing many disciplines and topics and, critically for this issue, being without universal definition. No widely recognized structured training exists to prepare medical students and residents for careers in this field, a problem compounded by difficulties finding knowledgeable mentors [3]. While barriers have been identified using quantitative surveys [2, 4], no qualitative studies have focused on describing barriers affecting physician careers in clinical cancer prevention. Conducting such studies can help surface and define unrecognized barriers for further quantitative evaluation with larger representative samples.

To provide such insight about career challenges and motivations of physicians in academic clinical cancer prevention and to identify opportunities to improve recruitment of medical students and early career physicians into the field, we mapped career pathways of physicians involved in academic cancer prevention at The University of Texas MD Anderson Cancer Center. Our goal was to identify barriers encountered and experiences that motivated them when considering and pursuing careers in cancer prevention. We also gathered recommendations to improve recruitment and define better pathways into clinical cancer prevention.

## Methods

This project was based at The University of Texas MD Anderson Cancer Center, a large NCI-designated comprehensive cancer center within the Texas Medical Center, Houston, Texas, with over 1700 clinical and nonclinical faculty members and a workforce of over 21,000 individuals. We identified participants through snowball recruitment starting with faculty within the five departments of the Division of Cancer Prevention and Population Sciences (DCPPS): behavioral science, clinical cancer prevention, epidemiology, health disparities research, and health services research. We identified other faculty at MD Anderson conducting research in cancer prevention who had served as mentors to trainees in the Cancer Prevention Research Training Program (CPRTP). After receiving approval from the Institutional Review Board of MD Anderson (IRB #2016-0397), we contacted prospective participants by email and follow-up calls. Participants were included based on the following criteria:

- Has a medical degree (MD, MBBS, etc.)
- Holds a faculty position at MD Anderson
- Is involved in cancer prevention research, defined as appointment in DCPPS, joint appointment in DCPPS, or mentorship of CPRTP trainees (e.g., bench research, population-based research/interventions, clinical activities)

Individuals were excluded according to the following criteria:

- Not faculty at MD Anderson or retired
- Did not complete medical training
- Not currently involved in cancer prevention activities (self-reported)

The recruitment email contained a link to a RedCap data collection form used to confirm eligibility, obtain electronic informed consent, and request times for scheduling in-person interviews.

This study was conducted using a constructivist grounded theory approach, described by Watling et al. [5]. Qualitative data were obtained during structured interviews with participants. Recorded interviews were coded and analyzed using specialized software, and themes were grouped to provide detailed insight into barriers and motivations for pathways to careers in cancer prevention. Participant recommendations for addressing challenges to cancer prevention career paths were also coded.

Participant curriculum vitae were obtained before interviews, so interviewers could familiarize themselves with the participants’ career achievements and academic record. Interviewers used an interview guide developed by the research team (SC, TT-D, OA) based on ideas drawn from studies of medical education and careers in cancer prevention (Table 1). Interviews ranged from 15 to 40 min and were audio-recorded with handwritten notes taken. Interviews were conducted by MK, JC, OA, and TT-D between July 2016 and October 2017. Data collection ended after all eligible faculty at MD Anderson had been contacted and willing participants had been interviewed by the study closing date, October 31, 2017.

**Table 1:** Interview guide

A. Career path decisions	
1. At what stage of your training did you first think about going into cancer prevention as specialty?	
2. What made you consider this path?	
3. During your training, did you ever participate in any formal or informal educational activity related to cancer prevention or academic medicine as a specialty choice? If any, what kind?	
4. Did you have a mentor during your training (medical school, graduate school, or residency)?	
5. Do you attribute your interest in academic medicine and cancer prevention (wholly or in part) to your mentor?	
6. Besides a mentor, were there other influential people (such as role models) who informed your decision to go into academic medicine/cancer prevention?	
B. Pathway to academic medicine/cancer prevention	
7. What other career choices were you considering?	
8. What made you choose one over the other?	
9. Looking back, would you have made different decisions knowing what you know now?	
C. Perceived barriers	
10. What were your major concerns when considering cancer prevention?	
11. Were there any difficulties understanding the responsibilities that a profession in cancer prevention research entitled? If any, how did you overcome those difficulties?	
12. What were some of the barriers to pursuing a career in academic medicine?	
13. Overall, would you say that you are satisfied with your choice to pursue academic medicine/cancer prevention? Please tell us why or why not	
D. Recommendations	
14. What could medical schools and/or residency programs do to increase interest in cancer prevention as a clinical or academic career among oncologists? (Open Box)	
15. Do you see merit in any of the initiatives below:	
• Sponsorship of mentored postdoctoral fellowships in cancer prevention	
• Development of a toolkit for training program directors	
• Provision of more educational sessions in cancer prevention (include natural products, behavioral interventions such as weight loss, tobacco cessation)	
• Special informational sessions for fellows on what a career in cancer prevention might look like	
16. Other comments	

### Data Analysis

Recorded interviews and notes were reviewed by MK, JC, and PQ for themes via the qualitative data management software, Atlas.ti (v7, 2015, Berlin, Germany). The coding template was developed by MK, JC, and PQ to address the study purpose: to map career pathways of physicians in cancer prevention, to identify factors that motivated pursuit of cancer prevention careers, barriers faced, and recommendations to address career challenges (Tables 2). Selected interviews were independently re-coded by different investigators to ensure complete capture of themes and ideas from each participant and alignment of coding by investigators.

**Table 2:** Qualitative themes data codebook

1. Career path decisions—academic medicine/cancer prevention	
Codes:	
• By design or by accident (was a conscious decision actually made)	
• When/at what stage	
• Strong influencing factor(s), event(s), or individual(s) that inspired the choice	
• Draw/motivation to specialize in cancer prevention/academic medicine	
2. Pathway to academic medicine/cancer prevention	
Codes:	
• How was your career direction determined?	
• Gaps in information/resources encountered	
• Opportunity cost of choosing cancer prevention (what options did you choose to ignore)	
3. Perceived barriers to recruiting students and residents into academic medicine/cancer prevention	
Codes:	
• Mentorship	
• Concerns (financial, professional, family time, prestige)	
• Misinformation	
• Hidden curriculum	
4. Recommendations	

## Results

Of 37 faculties eligible to participate, 21 declined participation, 18 were interviewed, but two withdrew, leaving data from 16 participants for analysis. Of these 16 participants, eight were women; five were self-designated as research faculty and the remaining 11 as clinical faculty although all participated in research activities. Two participants were assistant professors, six were associate professors, and eight were full professors.

### Impact of a Vaguely Defined Field (i.e., Cancer Prevention)

Given that the multidisciplinary, collaborative, and broadly diverse activities comprising cancer prevention make it challenging to clearly define the field, participants reported lack of clarity about cancer prevention as a barrier to knowing about careers in clinical cancer prevention (Table 3). In particular, several participants noted that they could not remember receiving education during medical school or residency about cancer prevention, whether due to lapses in memory or low emphasis placed upon cancer prevention. Many also cited lack of knowledge about cancer prevention practices as an impediment to applying cancer prevention to clinical care in their career paths.

**Table 3:** Themes and representative quotations from interviews with physician faculty in clinical cancer prevention

Themes and subthemes	Representative quotes
Vagueness of field impeded finding career path into cancer prevention for physicians	
Few role models for careers in clinical cancer prevention	“It was hard, when I finished my fellowship and joined the faculty, finding mentorship in this area was hard for me because I was interested in working with big population databases to see sort of what was happening more at the national level. And there were not really many people at [institution] that were doing that.”
Lack of widely known career resources and absence of structured training opportunities to guide entry into clinical cancer prevention careers	“Particularly for a clinician, that’s what I would say. It was more established for a population scientist or a behavioral scientist, but for an applied clinical cancer prevention, [it] did not really exist at the beginning of this in any mature form.”
	“Maybe I’ve just forgotten it all. But I do not think we really ever had that much training in cancer prevention type interventions or counseling. It’s much more focused on disease and how to treat disease.”
Uncertainty about how to connect cancer prevention to clinical career interests	“At that time, I really did not know anything about cancer prevention other than the cervix part of it, but I never thought of that as a field or a specialty. You know as I said even training here it did not even seem to be part of what we did.”
	“We know about cancer prevention, but you do not know that it’s a career...Especially when you are so highly specialized, in such highly specialized training, that you could sort of take all of that back to kind of a public health perspective, a cancer prevention perspective.”
Misinformation about cancer prevention	
Misattribution of responsibility of cancer prevention to other types of physicians	“The people who are most knowledgeable about cancer prevention are your primary care doctors. I did not think I wanted to be a primary care doctor so that wasn’t anywhere on my radar.”
	“I think one of the problems as specialists is we think that’s all the primary care provider’s area and that it does not really need us or we are not part of it.”
Discouraging negative comments about the field of cancer prevention and about pursuing cancer prevention careers	“My chair of the department there was like ‘I trained you to treat cancer, and you are trying to prevent what I told you to treat…’ ‘Oh, you are trying to put yourself out of business.’"
	“One of the barriers, is that the cancer prevention field has collectively seemed to have been too caught up and overlapped too much with the natural product research and that really trying to distance the cancer prevention field from the complementary alternative medicine field would probably help increase the external rigor that the field is perceived as.”
Motivations and pathways to clinical cancer prevention	
Unintended but inspiring exposure to cancer prevention careers and research	"So probably it would be I was already on faculty and kind of expanding research and had picked up a few projects that had some cancer prevention efforts. So it was probably ten years into my career on faculty."
Motivated by patient experiences	“It was just my clinical experience, and sort of like the frustration of seeing patients with cancer, with advanced stage disease that could not be cured that made me kind of want to look into how we can catch it early and how can we prevent it.”
Educational experiences that motivated active steps	“I then started attending some educational programs that [institution] was putting on Cancer Prevention and found that interesting. Tried to apply that in my practice when I was in private practice in the early years of my family medicine career. That was kind of the start of it all.”
	“Before I chose the PhD program at [institution] I have a long term goal to control cancer either by prevention or by blocking metastasis so both way, because in these two steps if we can do something good you can significantly decrease the mortality associated with cancer.”

In addition to low awareness and knowledge of clinical cancer prevention, participants also described the absence of visibly structured career paths and career resources as challenges to finding and successfully navigating careers in the field. They contrasted this deficiency with the general knowledge of clear paths that existed for other professionals into cancer prevention (e.g., NCI-funded cancer prevention postdoctoral research training programs) and into public health. This deficit, they commented, was especially apparent at early career stages when many participants were exploring their interests in cancer prevention. One participant observed that even within oncology training, strategies for incorporating a focus on cancer prevention were absent. Related to insufficient career resources, participants reported knowing few role models from the field and having difficulty finding knowledgeable mentors in cancer prevention for career and research guidance. Participants described challenges in finding mentors with particular expertise in population health, medical practice, and cancer prevention when they were beginning to pursue their interests in the field.

### Impact of Misunderstandings about Cancer Prevention

Not only was there not enough information about cancer prevention, but in some cases, the information was inaccurate. Some participants noted that they initially viewed cancer prevention as falling mainly within the purview of a primary care physician’s responsibility and did not understand how cancer prevention could align with their own career interests in oncology. Additionally, participants reported colleagues having negative comments and perceptions about the field of cancer prevention, which may have reinforced misunderstandings. Such views, some reported, may have perpetuated the misconception that cancer prevention as a field lacked scientific rigor. Indeed, one participant was told by a department chair that prevention was in opposition to training in oncology.

### Motivations and Pathways to Pursuing Cancer Prevention

In addition to barriers, participants described factors that facilitated their career pathways into cancer prevention. Many reported becoming involved in cancer prevention unintentionally while engaged in research projects related to cancer prevention, which led them to actively pursue their interests in the field. For example, some joined research projects with a cancer prevention aspect that helped them discover how this field aligned with their interests. Others had the fortunate opportunity to work with research mentors in cancer prevention, who provided feedback and guidance for career exploration in the field.

A recurring theme among participants was a desire to make a bigger difference in the lives of their cancer patients. Many physicians from multiple specialties expressed frustration about “missed opportunities” for cancer prevention as they saw the burden of advanced stage, incurable cancer diagnoses on their patients. This frustration, they reported to us, led them to turn towards cancer prevention activities, which gave them a greater sense of fulfillment and possibly more agency to intervene with their patients.

After committing to career interests in cancer prevention, participants took active steps to become more involved in the field. Participants pursued educational opportunities, research projects, and mentorship experiences to learn more about cancer prevention or to gain expertise in areas relevant to their interests. Learning through professional development courses about cancer prevention helped some see how such activities could be integrated in their work. Others pursued positions specifically in cancer prevention that allowed more time to do cancer prevention research or be involved in patient care directly focused on cancer prevention. Whether participants changed positions or not, they all began to include cancer prevention in their research and clinical practice after discovering the value of the field and their interests in it.

## Discussion

Advancing the current progress in cancer prevention contributed by physicians requires greater physician recruitment to the clinical cancer prevention workforce. Unfortunately, recruitment has been affected by multiple barriers and only some facilitators. Here, in-depth interviews with physicians at a major academic health center dedicated to cancer care and research provided rich insights about careers in cancer prevention beyond what has been obtained from quantitative surveys. Novel observations include the multiple adverse impacts of misconceptions about cancer prevention. Participants reported negative comments from influential colleagues and mentors that may have damaged working relationships or hindered pursuit of cancer prevention career. However, participants also reported experiences that motivated them to pursue such careers, including patient care experiences that deepened and made more personal the drive to provide better care through cancer prevention.

Some barriers that we report were previously reported but in less detail. For example, a task force to review workforce issues in cancer prevention research suggested that trainees do not realize that cancer prevention encompasses many disciplines and interests [6], supporting the idea that there is confusion about what is within the field of cancer prevention, as we found (Table 1). In particular, we found that clinical cancer prevention was often initially viewed as somebody else’s job, and some participants reported negative comments and perspectives from colleagues intended to discourage pursuit of these careers. Other themes our work echoed from earlier surveys of oncology fellows were that both the lack of clarity about careers in cancer prevention and the lack of clinical mentors in cancer prevention posed barriers to incorporating cancer prevention in their careers [2]. Specifically, some of our participants initially had difficulty connecting personal interests in cancer prevention with their desired careers. Even after establishing interest in cancer prevention, some still had difficulty finding suitable mentors and getting informed, knowledgeable, and supportive guidance about integrating cancer prevention successfully into their careers.

In addition to barriers to careers in cancer prevention, participants also described opportunities that stimulated interest and facilitated exploration of such careers. Most participants had an event or perspective that motivated them to seek more opportunities in academic cancer prevention. Many cited feeling frustrated by how little they could do for their cancer patients; but subsequently, through pursuit of a career in clinical cancer prevention, several expressed having great fulfillment through cancer prevention than in cancer treatment. Such sentiment, we speculate, reflected physicians’ deep empathy for the suffering of cancer patients—unnecessary if greater advances in preventing cancer could be achieved. Appealing both to such strong emotions—frustration and empathy—and to provider dedication to minimizing patients’ suffering could be ways to encourage interest in and pursuit of careers in clinical cancer prevention. Ideally this appeal would happen earlier in training, before accumulated frustration from missed opportunities for prevention stimulates a late shift to a career in cancer prevention.

Another participant suggestion to stimulate interest was involvement in cancer prevention research, whether as students or as physicians, because such experiences foster incorporation of research into physician careers [7]. Thus, the value of research experiences in cancer prevention for physicians-in-training is critical for learning how to conduct research in general, gaining direct experience conducting cancer prevention research, and expanding their knowledge of research topics in the field. These experiences may provide a space for physicians to solidify how their interests in cancer prevention apply to both research and clinical settings while building self-efficacy in cancer prevention research careers. Moreover, by working with scientists leading such projects, physicians-in-training have direct access to role models and mentors in the field.

As with all studies, ours has limitations. First, participants were selected from physicians holding faculty positions at a single institution within a division dedicated to cancer prevention, including a department of clinical cancer prevention, and who were required by inclusion criteria to be involved in cancer prevention activities. Therefore, individuals who had interest in cancer prevention but either never pursued those interests were formerly engaged in cancer prevention or had left the institution before our study began were not included. Also, physicians pursuing careers in clinical cancer prevention elsewhere may encounter different barriers and facilitators. Thus, we may not have captured in this initial effort all barriers to careers in academic clinical cancer prevention. As well, the physicians at the study institution may have attributes that make them resilient and persistent in their careers, producing a “healthy worker” bias, in which more workers who are “healthy” remain in the workforce and available for study inclusion. Regardless of the potential for such an effect, physicians we interviewed reported career barriers nonetheless and some were reported elsewhere [2, 6], suggesting that the experiences reported in our study were not uncommon and still have yet to be addressed successfully. A strength of our study was the diversity of participants by faculty rank and gender, such that the analysis of their interviews provided a rich and broad scope of career experiences in academic clinical cancer prevention. However, the results from this qualitative study do not represent the experiences of all physicians in academic cancer prevention. Additional work needs to assess how similar issues faced by those early in their careers today are to those experienced in early career by the senior faculty participants in our study as our study included only two individuals at the Assistant Professor rank. Nonetheless, these findings can support and guide further investigation using large groups of probability-sampled physicians from which conclusions can be generalized.

Given that we reported deeper insight into themes related to pursuing clinical cancer prevention careers than reported previously [2, 4, 6], the time to organize activities to improve recruitment into careers in clinical cancer prevention is past due. Steps can be taken in medical education, by funding agencies, and by national professional organizations to increase the visibility of the field, to reduce uncertainty and misinformation about physician careers in academic cancer prevention, and to address career barriers. For example, early and repeated career exposure in medical school and residency curricula by improving or emphasizing cancer prevention courses and curricular content could increase the visibility and importance placed on the field. This could simultaneously dispel misperceptions about clinical cancer prevention practice and offer strategies for weaving cancer prevention into clinical practice. Exposure can also occur through cancer prevention research, ideally funded for medical students and guided by seasoned cancer prevention scientists, both physicians and non-physicians working in multidisciplinary teams. These experiences, whether short-term summer experiences or fellowships, can deepen future physicians’ understanding of how to translate clinical practice needs in cancer prevention into systematic research that they can conduct and, someday, implement in the clinic and community for impact against cancer. For medical students entering clinical rotations, opportunities to rotate with physicians combining cancer prevention with clinical practice may have indelible impact upon their career trajectories into the field. Such early career exposure is critical for recruitment because students and residents need to know about cancer prevention first, before they can consider clinical cancer prevention as a career option. To sustain emphasis on the importance of cancer prevention during early career training, more questions about cancer prevention and control could be included by the Federation of State Medical Boards and National Board of Medical Examiners in licensing exams, as well as by the National Board of Osteopathic Medical Examiners in exams for those for in osteopathic medicine, thus requiring greater continuous attention while preparing for licensing.

National professional organizations, such as the American Association for Cancer Education (AACE), the American Association for Cancer Research (AACR), the American Society of Preventive Oncology (ASPO), and the American Society of Clinical Oncology (ASCO), are ideally positioned to facilitate mentorship and increase visibility of role models in cancer prevention. They could provide lists of members involved in cancer prevention research and available to mentor those exploring cancer prevention careers. Membership websites and meeting flyers about the different approaches and topics addressed in clinical cancer prevention could inform and attract individuals with interest in those areas. These organizations can also create position statements that challenge the hidden curriculum messages about clinical cancer prevention being a “lesser” pursuit than oncology and cancer treatment. Such messages will directly challenge misunderstandings and misperceptions about cancer prevention while making clear its valuable contribution to clinical and community practice, including primary prevention and early detection of cancer. These arguments must be made directly both to established colleagues and to those in training.

In sum, by understanding the career paths of successful physicians in the field, we have learned what events and experiences hindered and helped propel them into academic cancer prevention careers. Going forward with other studies that report the prevalence of career barriers and facilitators, this information can guide efforts to help others advance their career paths more directly and efficiently into the field, ideally earlier in their careers and by purposeful choice, not by accident, in isolation, or only after many years of effort. Only with such measures in place to improve recruitment into clinical cancer prevention will the cancer prevention workforce be able to achieve its full potential to lower the burden of preventable cancers on the public health. Indeed, prevention messages from physicians to encourage everyone to continue practicing behaviors that reduce cancer risk are important for the public health, including during times of difficulty, an important lesson from the recent COVID-19 pandemic.

## Data Availability

Data from coded interviews can be made available upon written request to and approval by the corresponding author and co-authors.
